# Revisiting the metal sites of nitrous oxide reductase in a low-dose structure from *Marinobacter nauticus*

**DOI:** 10.1007/s00775-024-02056-y

**Published:** 2024-05-08

**Authors:** Anja Pomowski, Simone Dell’Acqua, Anja Wüst, Sofia R. Pauleta, Isabel Moura, Oliver Einsle

**Affiliations:** 1 Institute for Biochemistry, Albert-Ludwigs-University Freiburg, Albertstrasse 21, 79104 Freiburg, Germany; 2 Dipartimento Di Chimica, Università Di Pavia, Via Taramelli 12, 27100 Pavia, Italy; 3 Microbial Stress Lab, UCIBIO—Applied Molecular Biosciences Unit, Department of Chemistry, NOVA School of Science and Technology, Universidade NOVA de Lisboa, 2829-516 Caparica, Portugal; 4 Associate Laboratory i4HB—Institute for Health and Bioeconomy, NOVA School of Science and Technology, Universidade NOVA de Lisboa, 2829-516 Caparica, Portugal; 5 LAQV, Department of Chemistry, NOVA School of Science and Technology, Universidade NOVA de Lisboa, 2529-516 Caparica, Portugal

**Keywords:** Nitrogen cycle, Nitrous oxide, N_2_O reductase, X-ray crystallography, Denitrification, Copper-containing enzyme

## Abstract

Copper-containing nitrous oxide reductase catalyzes a 2-electron reduction of the green-house gas N_2_O to yield N_2_. It contains two metal centers, the binuclear electron transfer site Cu_A_, and the unique, tetranuclear Cu_Z_ center that is the site of substrate binding. Different forms of the enzyme were described previously, representing variations in oxidation state and composition of the metal sites. Hypothesizing that many reported discrepancies in the structural data may be due to radiation damage during data collection, we determined the structure of anoxically isolated *Marinobacter nauticus* N_2_OR from diffraction data obtained with low-intensity X-rays from an in-house rotating anode generator and an image plate detector. The data set was of exceptional quality and yielded a structure at 1.5 Å resolution in a new crystal form. The Cu_A_ site of the enzyme shows two distinct conformations with potential relevance for intramolecular electron transfer, and the Cu_Z_ cluster is present in a [4Cu:2S] configuration. In addition, the structure contains three additional types of ions, and an analysis of anomalous scattering contributions confirms them to be Ca^2+^, K^+^, and Cl^–^. The uniformity of the present structure supports the hypothesis that many earlier analyses showed inhomogeneities due to radiation effects. Adding to the earlier description of the same enzyme with a [4Cu:S] Cu_Z_ site, a mechanistic model is presented, with a structurally flexible Cu_Z_ center that does not require the complete dissociation of a sulfide prior to N_2_O binding.

**Graphical Abstract:**

The [4Cu:2S] CuZ site in M. nauticus N 2O reductase. The electron density map shown is contoured at the 5 σ level, highlighting the presence of two sulfide ligands. 705x677mm (72 x 72 DPI)
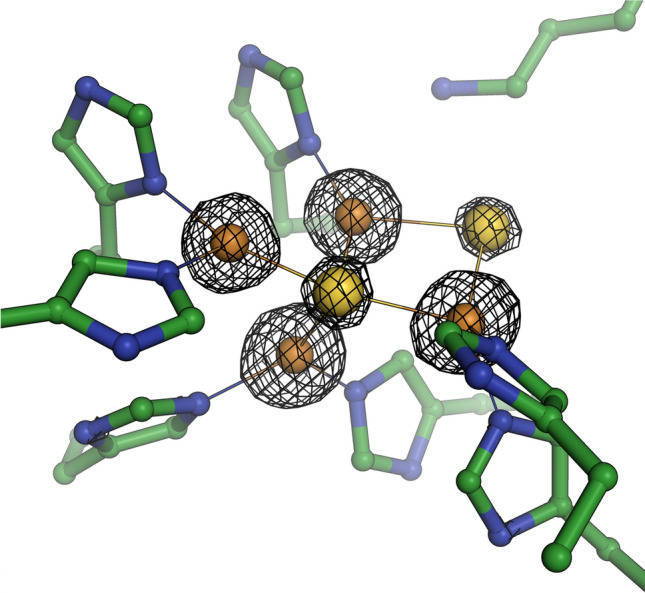

## Introduction

Bacterial denitrification is the dominant respiratory metabolic pathway in many microoxic or anoxic environments [1]. Denitrifying microorganisms assemble an electron transport chain in the cytoplasmic membrane that couples the stepwise reduction of nitrate (NO_3_^−^) via nitrite (NO_2_^−^), nitric oxide (NO), and nitrous oxide (N_2_O) to dinitrogen (N_2_) to the generation of a proton motive force [2]. The final step of the pathway is a 2-electron reduction of N_2_O catalyzed by the copper-containing enzyme nitrous oxide reductase (N_2_OR) [3–5]. The process of fertilization in industrial agriculture creates nitrate-rich habitats that support the growth of denitrifying microbes to compete with crop plants, and N_2_OR then often is inactivated and gaseous N_2_O is released as a metabolic end product [6], resulting in a steady rise in atmospheric levels of nitrous oxide, in lockstep with the increasing fertilizer use during the past century [7]. With a global warming potential that exceeds the one of CO_2_ 300-fold, nitrous oxide is a potent green-house gas, and it has an additional deleterious effect as an ozone-depleting substance. Its increasing concentration in the atmosphere has thus prompted its designation as the most critical anthropogenic emission in the twenty-first century [8].

*Marinobacter nauticus*, formerly known as *Pseudomonas nautica* or *Marinobacter hydrocarbonoclasticus* [9], is a denitrifying, marine Gammaproteobacterium that forms biofilms on hydrophobic organic matter but can also sustain a pelagic lifestyle [10]. Its N_2_OR is a 130 kDa homodimer encoded by the *nosZ* gene that contains two copper sites, Cu_A_ and Cu_Z_ [11]. Cu_A_, a dinuclear, mixed-valent [Cu^1.5+^:Cu^1.5+^] center, transfers a single electron with a midpoint redox potential of + 65 ± 5 mV, at pH 7.6 [12]. It is located in the C-terminal cupredoxin domain of NosZ, similar to the Cu_A_ site in cytochrome *c* oxidase [13]. The N-terminal domain of NosZ, with a 7-bladed β propeller fold, holds the Cu_Z_ center, a tetranuclear copper cluster that is unique to N_2_OR. The enzyme from *M. nauticus* was the first to be characterized by X-ray crystallography, and an initial description of Cu_Z_ as a [4Cu:O] center [14] was subsequently revised to a [4Cu:S] stoichiometry [15], in line with available spectroscopic data [5, 16] and with further structural analyses of the enzymes from *Paracoccus denitrificans* [17] and “*Achromobacter cycloclastes*” [18]. In these structures, Cu_Z_ is a µ_4_-S-bridged Cu cluster with a total charge of +3, i.e., formal copper oxidation states of [1Cu^2+^:3Cu^+^] [3]. This state is characterized by a single band at 650 nm in electron excitation spectroscopy, arising from an S → Cu LMCT transition resulting in a blue color of the enzyme [19]. This simple spectrum is commonly masked by the more complex signature of the mixed-valent Cu_A_ yielding a pink enzyme, but can be revealed when the binuclear site is reduced to a colorless all-cuprous state with ascorbate [20]. The pink form of N_2_OR has been designated form II [5] and was obtained as the oxidized state of the enzyme when prepared in the presence of dioxygen [17, 18, 20]. A spectroscopically distinct, purple form I was first described for *Pseudomonas stutzeri* [21], but was later also found in *Achromobacter xylosoxidans* [22] and *M. nauticus* [23]. It shows an absorption maximum at 538 nm, and while it can also be reduced with ascorbate to a blue form, this resulting species consists of two bands at 552 nm and 650 nm and is thus distinct from the one described above [4]. In 2011, the purple N_2_OR from *P. stutzeri* was crystallized under anoxic conditions [24], revealing that here the enzyme contains a Cu_Z_ site of composition [4Cu:2S] [25]. The second sulfur atom in this site, S_Z2_, is labile and seems to be depleted—to varying degrees—in different batches of protein. This revised stoichiometry of the tetranuclear site explained the spectroscopic findings and clarified that the Cu_Z_ site of purple N_2_OR form I is a [4Cu:2S] cluster, while the desulfurated [4Cu:S] cluster of the pink form II enzyme was assigned as the Cu_Z_* species defined previously by spectroscopy [4, 5]. In purple N_2_OR as isolated, Cu_Z_ is present in a [2Cu^2+^:2Cu^+^] state that cannot be reduced with ascorbate but converts to a [1Cu^2+^:3Cu^+^] state upon incubation with dithionite [3]. This state is distinct from the one found for Cu_Z_* with respect to its EPR spectra. Based on a recombinant production system for active N_2_OR in *E. coli* that yielded catalytically active, purple N_2_OR with a [4Cu:2S] Cu_Z_ site [26], the systematic replacement of the seven histidine ligands to Cu_Z_ led to a complete loss of metalation in six cases, but to an unexpected spectrum resembling a form II enzyme in the case of the H382A variant [27]. However, rather than the [4Cu:2S] Cu_Z_ cluster commonly associated with this spectrum, the crystal structure of H382A N_2_OR contained a [3Cu:2S] center that was catalytically inactive but retained three copper ions liganded by sulfides, and it still produced the CT band at 650 nm. Interestingly, sulfide S_Z2_ was present in this cluster, but in the absence of the Cu_Z1_ ion it bound in the native enzyme, it had shifted its position toward a nearby lysine. The implication was that the difference between the reported Cu_Z_ and Cu_Z_* sites may not be the complete loss of sulfide S_Z2_ [28], but rather its dissociation from Cu_1_ that also leads to the loss of the 552 nm CT band, rendering the second sulfide invisible in UV/Vis spectra. If so, this may reconcile different hypotheses about the mode of N_2_O binding to the Cu_Z_ site and the mechanism of its reduction, as a mechanistic proposal for N_2_OR had suggested substrate binding to Cu_1_ and Cu_4_ of Cu_Z_ [29–31], which seemed incompatible with the presence of S_Z2_, but may well be feasible if the shift of S_Z2_ would render both Cu_1_ and Cu_4_ three-coordinate [27].

Considering these results, we revisited *M. nauticus* N_2_OR, whose initial analysis had a [4Cu:S] Cu_Z_ site with H_2_O bound at the Cu_1_–Cu_4_ edge (PDB 1QNI), providing the basis for the proposed mechanism [29]. The positive midpoint potential of copper sites in proteins makes them particularly prone to photoreduction or even more severe forms of radiation damage during diffraction data collection at high-flux X-ray sources, such as synchrotrons. We therefore analyzed the anoxically isolated, purple form of *Mn*N_2_OR using the far lower photon flux of a rotating anode X-ray generator while retaining cryogenic conditions during data collection, aiming to generate a diffraction data set that was unperturbed by radiation-induced effects and more accurately represented the enzyme in its active form as isolated from its native host.

## Experimental procedures

### Cell growth and protein purification

*M. nauticus* was grown under denitrifying conditions in the presence of nitrate, as described elsewhere [16]. Cell disruption was carried out in an anoxic chamber with an N_2_/H_2_ (95%/5%) atmosphere (Coy Labs), where H_2_ was used to react with residual oxygen on a platinum catalyst surface to maintain pO_2_ < 1 ppm. During protein purification, samples, buffers, and columns were kept anoxic using modified Schlenk techniques. Approximately 180 g of cells (wet weight) were resuspended in 10 mM Tris/HCl buffer at pH 7.6 and broken in a microfluidizer (microfluidics). From the resulting crude extract, cell debris and membranes were sedimented by centrifugation at 100,000×*g* for 1 h. With the supernatant, all steps were carried out without breaks to avoid storage of the sample or freeze–thaw cycles. The soluble fraction was loaded onto a DEAE-FF anion exchange column (GE Healthcare) equilibrated with 10 mM Tris/HCl buffer at pH 7.6 and washed with the same buffer until A_280nm_ reached baseline. The column was developed with a linear gradient from 10 to 500 mM Tris/HCl buffer, pH 7.6, from which N_2_O reductase eluted at approximately 300 mM Tris/HCl. The pooled fractions of the elution peak were then dialyzed against 10 mM Tris/HCl buffer at pH 7.6, and subsequently loaded onto a second anion exchange column (HiTrapQ, GE Healthcare) that was equilibrate in the same buffer. The column was again washed to baseline and developed with a linear gradient from 10 to 500 mM Tris/HCl buffer, pH 7.6, with elution of N_2_OR at approximately 300 mM Tris/HCl. As a final step, the eluate was loaded onto a Superdex 200 size exclusion column (Cytiva) equilibrated with 300 mM Tris/HCl buffer at pH 7.6. The pure enzyme eluted as a symmetric peak and was concentrated by ultrafiltration (50 kDa MWCO, Sartorius) to 14 mg mL^−1^, while at the same time, the buffer was exchanged to 10 mM Tris/HCl, pH 7.6, for crystallization. Protein was determined using the bicinchoninic acid method [32], using bovine serum albumin as protein standard.

### Crystallization and data collection

*Mn*N_2_OR was crystallized by sitting drop vapor diffusion in an anoxic glove box (Coy Labs) at 303 K, in an N_2_/H_2_ atmosphere with < 1 ppm O_2_. 2 µL of protein solution (14 mg mL^−1^) were mixed with 2 µL of a reservoir solution containing 15% (*w/v*) polyethylene glycol 8000, 0.6 M NaCl, and 0.1 M imidazole/malate buffer at pH 7.2 and equilibrated against 50 µL of the same reservoir solution in a sealed crystallization plate (SwissSci). Large plate-like crystals were obtained in less than 1 day. For data collection, a crystal was transferred into a harvesting buffer that consisted of the reservoir solution with 15% (*v/v*) of 2*R*,3*R*-butane diol as a cryoprotectant. The crystal was then flash-cooled in liquid nitrogen before being removed from the anoxic chamber. Diffraction data were collected on a rotating copper anode X-ray generator (Rigaku MicroMax 007HF with a marresearch mar345dtb image plate system) at a wavelength of λ = 1.5418 Å and a temperature of 100 K.

### Structure solution and refinement

The data set was integrated with XDS [33] and scaled with AIMLESS [34] from the CCP4 suite [35]. Molecular replacement with MOLREP [36] was used to locate two copies of the dimeric enzyme in the asymmetric unit, using the previous structure of *Mn*N_2_OR (PDB 1QNI) as a search model. Model building was conducted in COOT [37], and REFMAC5 [38] was used for refinement. The quality of the final model was evaluated with MolProbity [39]. Data collection and refinement statistics are summarized in Table 1.

**Table 1:** Data collection and refinement statistics

Data set	
Space group	*P* 1
Cell constants *a, b, c* [Å] *α, β, γ* [°]	65.2, 69.4, 153.1 81.4, 77.9, 88.6
Wavelength [Å]	1.5418
Resolution limits [Å]	23.49 – 1.50 (1.52 – 1.50)
Completeness (%)	91.4 (83.4)
Total number of observations	757,181 (33,806)
Unique reflections	381,570 (17,344)
Multiplicity	2.0 (1.9)
*R*_merge_^**a**^	0.026 (0.258)
*R*_p.i.m_ [62]	0.026 (0.258)
Mean I/σ(I)	12.1 (3.0)
CC_1/2_ (outer shell) [63]	0.870
Refinement statistics	
*R*_cryst_^**b**^	0.135 (0.294)
*R*_free_	0.158 (0.322)
Non-hydrogen atoms excl. solvent	18,482
Solvent molecules	3,222
Cruickshank’s DPI (Å) [64]	0.036
Wilson B-factor (Å^2^)	45.6
Avg. B-factor protein (Å^2^)	69.2
Avg. B-factor cofactors (Å^2^)	59.0
Avg. B-factor solvent (Å^2^)	56.2
r.m.s. deviations from ideal values	
Bond lengths (Å)	0.005
Bond angles (º)	1.10
Ramachandran statistics	
Preferred regions	2072 (93.8%)
Allowed regions	117 (5.3%)
Outliers	19 (0.9%)

^*a*^$$R_{merge} = {\mkern 1mu} \Sigma_{hkl} \left[ {\left( {\Sigma_{i} |I_{i} - {\mkern 1mu} I|} \right){\mkern 1mu} /{\mkern 1mu} \Sigma_{i} I_{i} } \right]$$

^*b*^$$R_{{{\text{cryst}}}} = \, \Sigma_{hkl} \left| {\left| {F_{{{\text{obs}}}} } \right| \, - \, } \right|F_{{{\text{calc}}}} \left| {\left| { \, / \, \Sigma_{hkl} } \right|F_{{{\text{obs}}}} } \right|$$

*R*_free_ is the cross-validation *R* value for a test set of 5 % of unique reflections

^**c**^Ramachandran statistics as defined by PROCHECK [65]

## Results and discussion

Diffraction data from a plate-like single crystal of *Mn*N_2_OR were collected using Cu K_α_ radiation (*λ* = 1.5418 Å) on a rotating anode X-ray generator with a mar345dtb image plate detector at a temperature of 100 K. The crystal belonged to the triclinic space group *P*1 and diffracted beyond the detector edge at the minimal possible crystal-to-detector distance, yielding data to 1.5 Å resolution with a mean *I*/σ(*I*) of 2.6 in the highest resolution shell (Table 1). The data set proved to be of outstanding quality and the excellent electron density map compared favorably to the best available data obtained from synchrotron sources, while presumably not suffering from radiation-induced changes in the architecture of the metal sites. Notably, the quality of the map also far exceeded that of the earlier, synchrotron-derived structure from the same organism that was refined to 2.4 Å resolution (PDB 1QNI) [14, 15]. This crystal form contained two N_2_OR dimers in the asymmetric unit and thus four independent observations of each feature.

### Overall structure

**Fig. 1:**
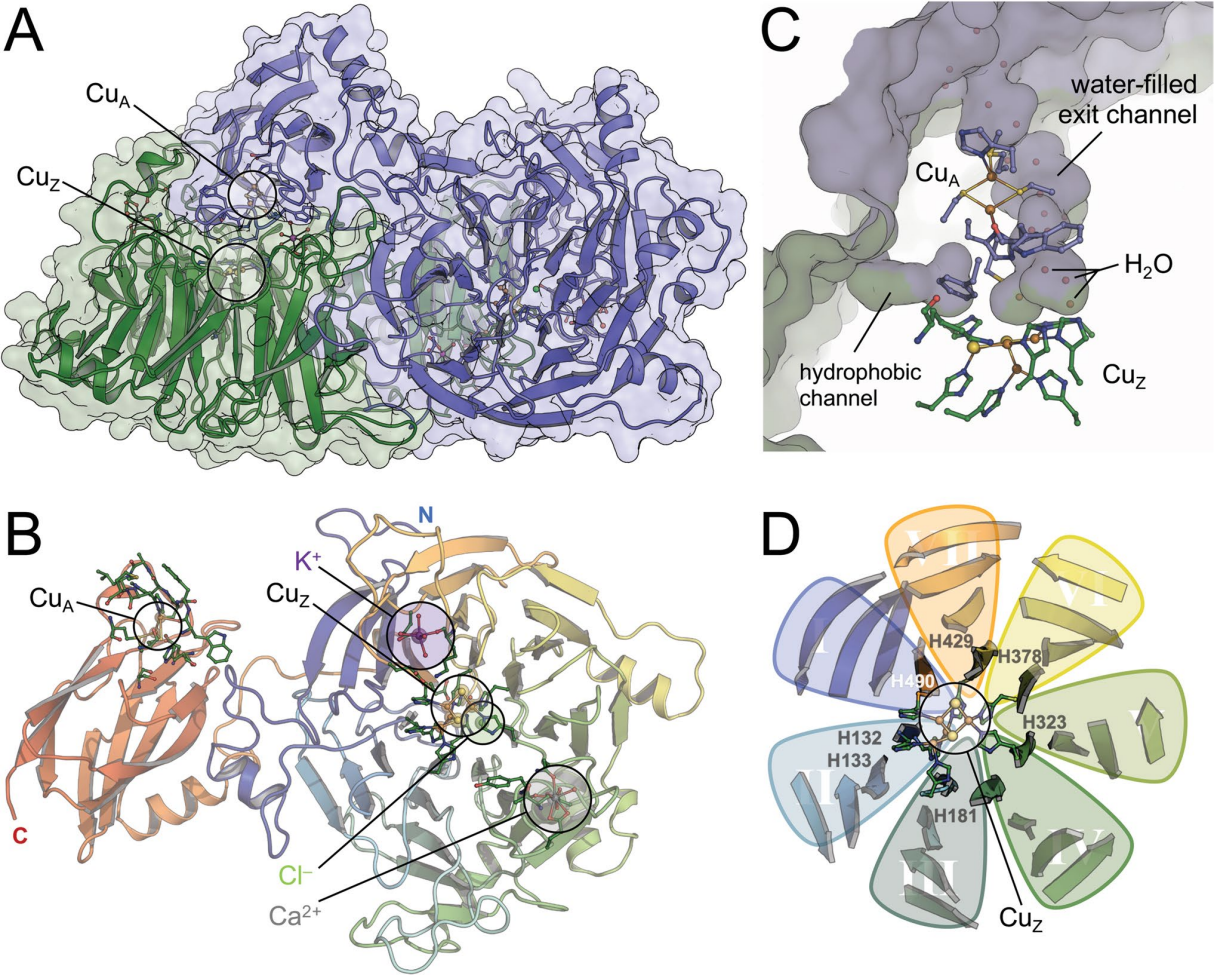
Low-dose structure of *M. nauticus* N_2_OR. **A** The head-to-tail homodimer of N_2_OR with protomer A in green, protomer B in blue. The Cu_A_ site of one protomer forms a combined active site with the Cu_Z_ site of the other. **B** Cartoon representation of a NosZ monomer with the distinct two-domain architecture, colored from blue at the N-terminus to red at the C-terminus. The N-terminal domain forms a seven-bladed β-propeller with Cu_Z_ at its center, while Cu_A_ is bound to the C-terminal domain, which attains the cupredoxin fold. The positions of the ions discussed in the text are indicated. **C** A hydrophobic channel along the dimer interface allows for access of the substrate N_2_O and egress of product N_2_, while the second product water is released into a water-filled exit channel that leads back to the protein surface. **D** The seven blades of the N-terminal domain and Cu_Z_ at the hub of the propeller. Except for blade IV, each blade contributes a histidine ligand to Cu_Z_. Blade II contributes two consecutive histidines, to a total of seven ligands to the four copper ions of the site

The low-dose structure of *Mn*N_2_OR aligns with the previous model with a root-mean-squared deviation (r.m.s.d.) of 0.25 Å for 7727 atoms [14, 15]. The additional level of detail offered by the improved resolution allows for a detailed comparison with structures of the enzyme from other organisms. *Mn*N_2_OR forms the typical head-to-tail homodimer (Fig. 1A), with the N-terminal, seven-bladed β-propeller domain of one monomer in close contact with the C-terminal cupredoxin domain of the other (Fig. 1B). At the interface of both domains, the Cu_A_ site closely approaches the Cu_Z_ cluster of the other subunit to form a combined active site that is accessible via a hydrophobic substrate channel running along the subunit interface, with a water-filled cavity behind the Cu_Z_ cluster (Fig. 1C). These features were previously observed in a structure of *Ps*N_2_OR prepared under anoxic conditions after pressurization with N_2_O gas, in which the substrate N_2_O was bound between the two copper sites [25]. The gaseous substrate N_2_O and product N_2_ enter and exit via this hydrophobic channel, while the second product, H_2_O, can exit through the water-filled cavity [4].

### The electron transfer site Cu_A_

**Fig. 2:**
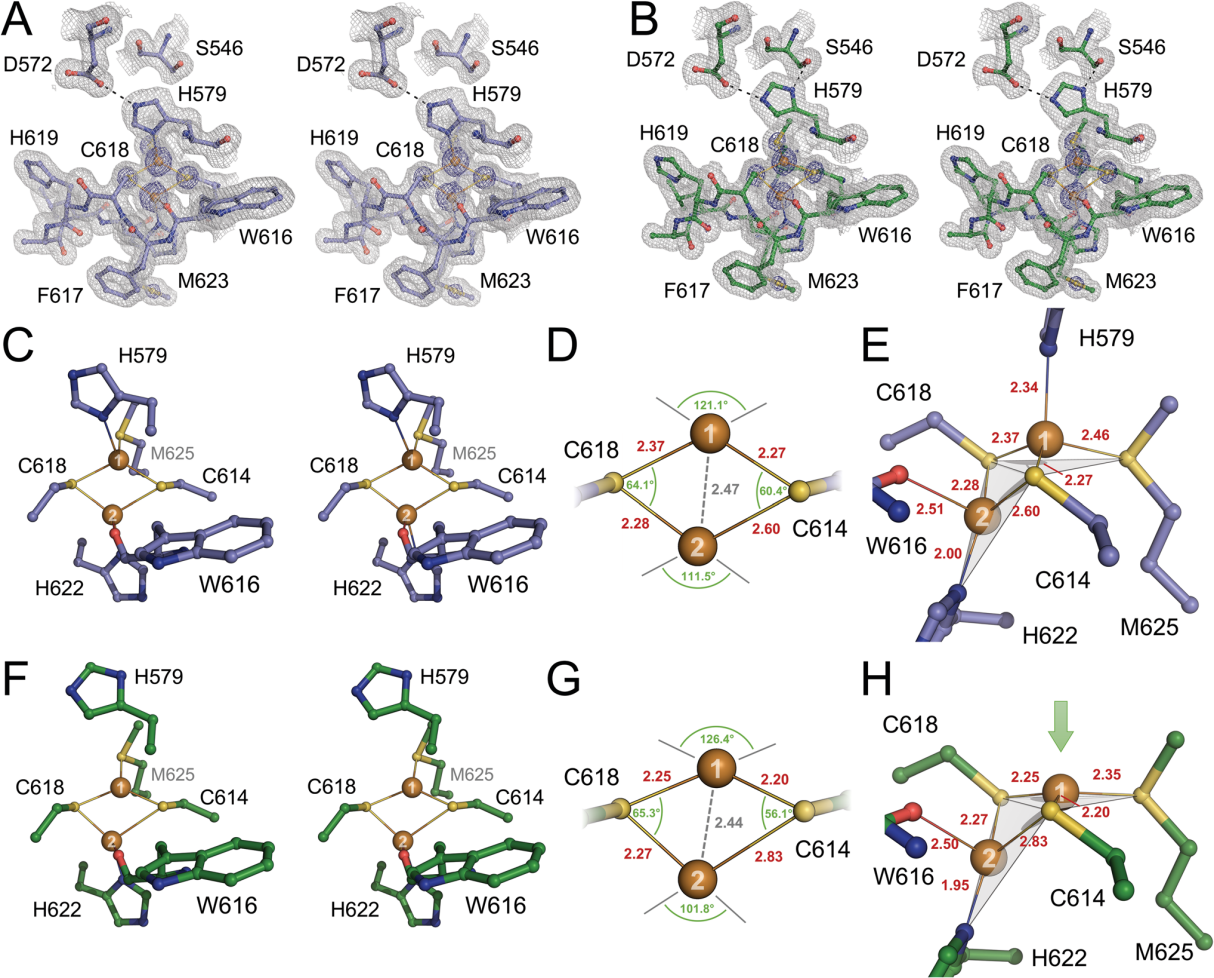
The Cu_A_ site in *Mn*N_2_OR. **A** 2*F*_o_–*F*_c_ difference electron density map around the Cu_A_ site in protomer A, contoured at the 1σ (grey) and 5σ (blue) levels. Orientation as in (**C**). **B** The corresponding representation for the Cu_A_ site in protomer B. **C** Cu_A_ in protomer A in the IN conformation of H579 with the ligands of the first coordination sphere. **D** Bond distances (in Å) and angles in the 2Cu:2S diamond core of Cu_A_ in protomer A, showing distortion from an ideal geometry, in particular in a long bond from Cu_A2_ to the S_γ_ atom of C614. **E** Contrary to [2Fe:2S] clusters, the ligand field of the copper ions is not very close to tetrahedral. While Cu_A1_ remains largely tetrahedral, with an extended bond to M625, the environment of Cu_A2_ is better described as trigonal pyramidal, with the backbone carbonyl of Q616 as an axial ligand and the Cu ion in the plane of the other three, C614, C618, and H622. **F** Cu_A_ with H579 in the OUT-conformation in protomer B, rendering Cu_A1_ three-coordinate. **G** The 2Cu:2S core now shows much stronger distortion, with the Cu_A2_–C614-S_γ_ bond elongated to 2.83 Å. **H** With the release of H579, Cu_A2_ moves almost fully into the plane of the remaining ligands, leading to a trigonal planar coordination environment. Cu_A2_ remains largely unchanged. Panels (**A**), (**B**), (**C**), and (**F**) are rendered for wall-eyed stereo viewing

Originally described as one of the two spectroscopically distinct copper sites in respiratory cytochrome *c* oxidase, the exact nature of Cu_A_ remained under debate (‘embattled’, in the words of Helmut Beinert) for years [40, 41]. In EPR, Cu_A_ appeared with an unusually small hyperfine splitting around *g*_⊥_ = 2.18 that in most cases was not even resolved into what was expected to be a four-line pattern for a mononuclear copper, and the presence of hemes *a* and *a*_3_ in the oxidase further obfuscated the signature of the copper site. In 1989, Dooley and Zumft showed that *Ps*N_2_OR also contains a Cu_A_-type site that is not masked by the presence of heme, and after some exchange on the nature of Cu_A_ [42, 43], Kroneck, Zumft, and others established along various lines the dinuclear, valence-delocalized nature of the site even before a first crystal structure of cytochrome *c* oxidase became available [44, 45]. In structures of cytochrome *c* oxidases, N_2_O reductases and diverse natural and engineered Cu_A_ domains [19, 46–48], two Cu ions form a central rhomb with the thiols of two cysteines, and the distorted tetrahedral ligand environment of the coppers is completed by a histidine at each metal, plus a methionine for Cu_A1_, the ion more distal from the Cu_Z_ cluster (Fig. 2), and a backbone carbonyl for Cu_A2_. The same arrangement was found and modeled in both Cu_A_ sites within the *Mn*N_2_OR dimer (Fig. 2A, B), with C614 and C618 as bridging cysteines, H579 and M625 as ligands to Cu_A1_, and H622 and the backbone carbonyl of W616 as ligands to Cu_A2_ (Fig. 2C). The initial analysis of *Mn*N_2_OR had established these residues and modeled all six occurrences of Cu_A_ in the asymmetric unit in the same conformation [14]. This uniform nature of Cu_A_ was first challenged with the analysis of *Ps*N_2_OR under anoxic conditions [25], where the corresponding Cu_A_ sites now attained two distinct conformations. Typically, in one of the two copies of the monomer, the histidine ligand to Cu_A2_ (H583 in this organism) had its imidazole moiety rotated away from Cu_A1_ by 130° to form a short hydrogen bond to a nearby serine (S550). In the following, this will be designated the OUT-conformation. Only in structures with bound N_2_O, this histidine was consistently flipped back to the copper (the IN conformation), and we hypothesized at the time that this may represent a mechanism to gate electron transfer during catalysis [25]. Upon establishment of a recombinant production system for a functional *Ps*N_2_OR in *E. coli*, [26] the metal-replete form I structure had both Cu_A_ sites in the OUT-conformation at Cu_A1_. For the low-dose structure reported here, generated from protein isolated from actively denitrifying *M. nauticus*, the conformations of the Cu_A_ sites were therefore of particular interest.

The two N_2_OR dimers of the asymmetric unit indeed differed in the conformation of residue H579, which was a ligand to Cu_A1_ in the A protomer of both dimers (IN conformation) (Fig, 2A, C), but rotated toward S546 in protomer B (OUT-conformation) (Fig. 2B, F). These positions corresponded to the two conformations observed in the first *Ps*N_2_OR structures, and the precise definition of both sites now allowed to resolve distortions and asymmetries of the metal centers. In the IN conformation of protomer A, the Cu_2_S_2_ rhomb was already slightly distorted: At a short Cu–Cu distance of 2.47 Å (compared to 2.5 Å in the earlier analysis with synchrotron data at 2.4 Å resolution [14]), the Cu_A2_–Sγ-C614 distance was extended to 2.6 Å, and the Cu_A1_–Sγ-C618 distance was slightly elongated to 2.37 Å. This conformation is more similar to all other reported Cu_A_ sites except for *P. stutzeri*. Cu_A1_ had a nearly ideally tetrahedral ligand field with three sulfurs and a nitrogen, and all bond distances between 2.27 Å and 2.46 Å (Fig. 2E). The latter distance to the S_δ_ of M625 was far shorter than for typical methionine ligands in type I copper centers [49, 50]. In contrast, the geometry of Cu_A2_ diverged farther from an ideal tetrahedron, with the metal residing almost exactly in the plane of ligands C614, C618, and W616 (Fig. 2E), implying that it is better described as trigonal pyramidal. Interestingly, the second longest bond distance after the one to C618 was 2.51 Å to the backbone carbonyl of W616, suggesting a rather weak Cu–O bond. When in protomer B residue H579 rotated into the OUT-conformation (Fig. 2F), the resulting geometrical distortion of the Cu_2_S_2_ core was quite significant (Fig. 2G). With slight changes in distances and angles, the rhomb became more asymmetric, and the Cu_A2_–Sγ-C614 bond weakened further, its length increasing to 2.83 Å. The Cu–Cu distance contracted only slightly to 2.44 Å. The most obvious difference, however, was geometric. While the environment of Cu_A2_ changed only with respect to C618, the missing H579 now caused Cu_A1_ to move almost fully into the plane of C614, C618, and M625, so that the coordination geometry became trigonal planar, while the environment of Cu_A2_ remained trigonal pyramidal (Fig. 2H). For *Ps*N_2_OR, we previously suggested that H579 serves as a gatekeeper for electron and proton transfer in the enzyme [25, 51, 52], and the distortions of Cu_A_ observed here very likely also reflect in distinct changes of the midpoint potential of the site. Furthermore, the methionine ligand to Cu_A1_ was frequently designated as a weak axial ligand at this position, and the influence of the protein matrix was suggested to keep it bound, while at the same time lowering the midpoint redox potential [53]. More recently, an NMR analysis revealed significant spin density on the methionine, in line with a stronger binding and an involvement in electron transfer [54]. The present analysis adds an additional level of complexity to the electronic properties of Cu_A_, in that the methionine ligand to Cu_A1_ remains bound to the metal in both states but undergoes changes both in bond length and geometry.

### Cu_Z_ is a [4Cu:2S] cluster

**Fig. 3:**
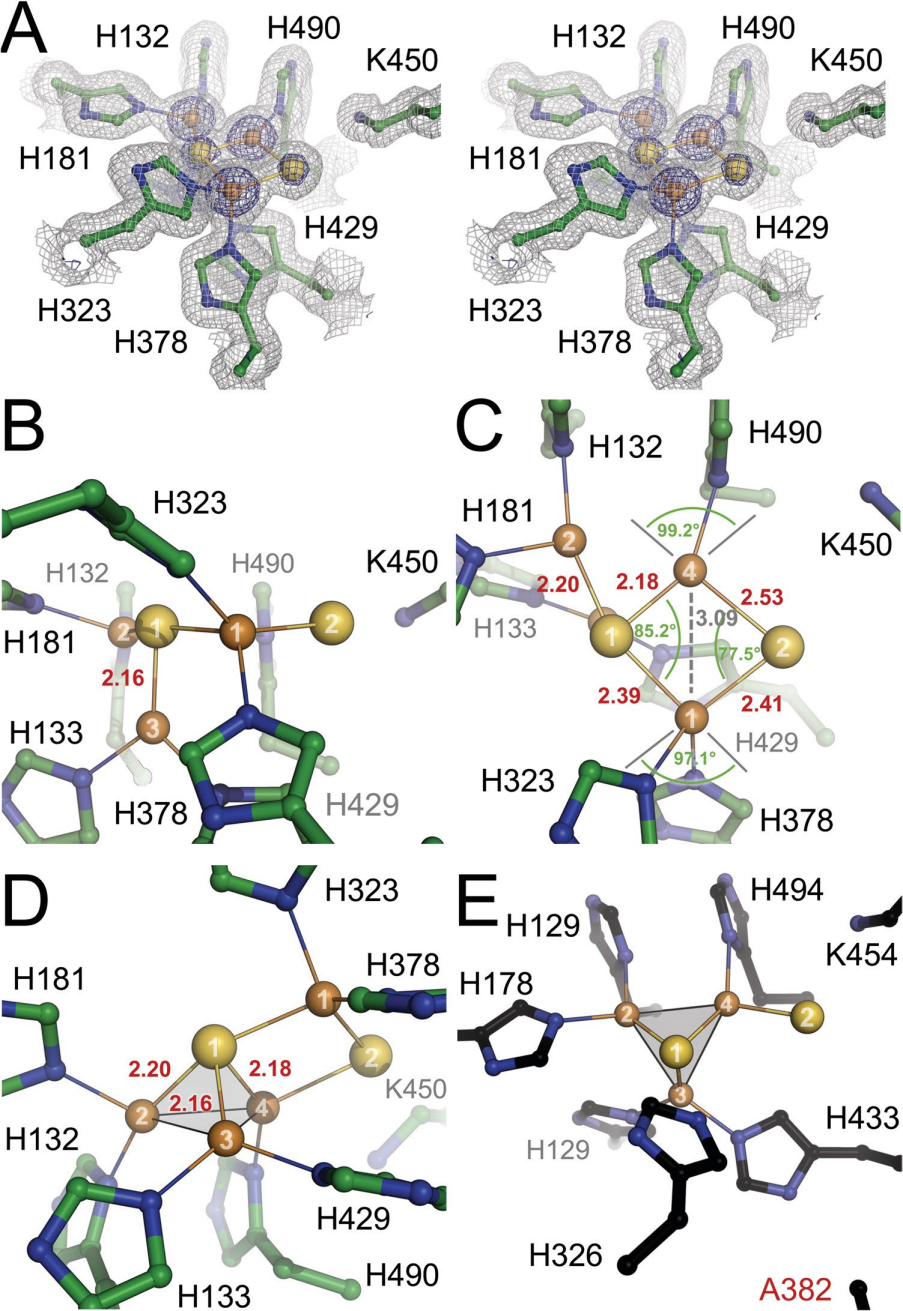
The [4Cu:2S] Cu_Z_ site in *Mn*N_2_OR. **A** Stereo image of a 2*F*_o_-*F*_c_ difference electron density map around the Cu_Z_ site in protomer B, contoured at the 1σ (grey) and 5σ (blue) levels. **B** Front view of Cu_Z_, highlighting the plane formed by Cu_Z1_, Cu_Z2_, Cu_Z4_ and the two sulfides. **C** Top view of Cu_Z_, showing the Cu_2_S_2_ rhomb of Cu_Z1_ and Cu_Z4_ and the two sulfides, with distances in Å an angles. **D** Sulfide S_Z1_ coordinates all four copper ions. It forms a tetrahedron with short bond distances to Cu_Z1_, Cu_Z2_, and Cu_Z3_, with short Cu–Cu distances indicative of metal–metal bonding. **E** In an H382A variant of *Ps*N_2_OR, the removal of histidine 382 led to the loss of Cu_Z1_. The remainder of Cu_Z_, with the now dangling S_Z2_ and the Cu_3_S tetrahedron remained largely unchanged [27]

Adjacent to Cu_A_ but in the other protomer of the N_2_OR dimer, Cu_Z_ is located at the hub of the N-terminal seven-bladed β-propeller domain, coordinated by seven histidine ligands: H132, H133, H181, H323, H378, H429, and H490. The fourth propeller blade that also contains the Ca^2+^-binding loop (vide infra) is the only one that does not contribute a ligand to Cu_Z_, while the second blade provides two. The first blade also provides a histidine, although its innermost β-strand is contributed by the C-terminus of the domain (Fig. 1D). Contrary to the IN- and OUT-conformations of the Cu_A_ sites, the four individual observations of Cu_Z_ were indistinguishable in the low-dose structure, where Cu_Z_ consistently was a [4Cu: 2S] site, with a largely symmetric Cu_2_S_2_ diamond core that is reminiscent of Cu_A_ (Fig. 3A). With four metal ions, two sulfides, and seven histidine ligands, Cu_Z_ is a uniquely asymmetrical metal site, and the architectures reported in previous structure determinations varied substantially, in particular with respect to the Cu_Z1_–Cu_Z4_ edge of the cluster [51]. In the present structure, Cu_Z_ seemed unperturbed, which was also reflected in a perfect alignment of all seven histidines with the coordinated coppers, which was not always the case in previous structures (Fig. 3A). Five of the six ions of Cu_Z_ were almost perfectly in a plane, with only Cu_Z3_ outside at 2.16 Å distance (Fig. 3B). Each Cu ion was liganded by two histidines, with the exception of Cu_Z4_, to which H490 was the only protein ligand (Fig. 3B,C). The Cu_2_S_2_ rhomb in Cu_Z_ is formed by Cu_Z1_, Cu_Z4_ and the two sulfides S_Z1_ and S_Z2_. The Cu–S bonds to S_Z1_ were shorter than those to S_Z2_, and the Cu_Z1_–Cu_Z4_ distance of 3.1 Å was substantially longer than the metal–metal distance in Cu_A_ (Fig. 3C). Due to this, the angles in this rhomb were closer to orthogonal than in Cu_A_. Arguably, the most unusual feature of this unique cluster is the arrangement of four Cu ions around the central sulfide S_Z1_. Three of the metal ions, Cu_Z2_, Cu_Z3_, and Cu_Z4_, form a nearly equilateral triangle that is completed to a tetrahedron by S_Z1_, and the bond distances in this unit are remarkably short. The three copper ions had distances of 2.16 ± 0.03 Å to S_Z1_, and the distances of 2.61 ± 0.01 Å for Cu_Z2_–Cu_Z4_, 2.64 ± 0.01 Å for Cu_Z2_–Cu_Z3_, and 2.81 ± 0.01 Å for Cu_Z3_–Cu_Z4_ are indicative of metal–metal bonding. They fall in the range of distances also observed in Cu_A_ sites in other analyses, while these distances in Cu_A_ are shorter in the present structure (Fig. 2D, G). Notably, this tetrahedral structure was retained in a H382A variant of recombinant *Ps*N_2_OR [27], although Cu_Z1_ was lost due to the removal of the histidine that corresponds to H378 in *Mn*N_2_OR (Fig. 3E). In this structure, even the second sulfide S_Z2_ was still present, now as a dangling ligand with a long hydrogen bond to K545 (K450 in *Mn*N_2_OR). It may well be the stability of this tetrahedral core that allows for the flexibility of sulfide S_Z2_, as observed in previous analyses.

In the synchrotron data structure of *Mn*N_2_OR, the position of sulfide S_Z2_ was modeled as a water molecule bridging Cu_Z1_ and Cu_Z4_ [14, 15]. However, the position of this ligand coincided very well with S_Z2_, and the six observations of Cu_Z_ in the asymmetric unit of these crystals yield bond distances of 2.7 ± 0.2 Å to Cu_Z1_ and 2.3 ± 0.1 Å to Cu_Z4_ (stating only a single decimal at 2.4 Å resolution). These values agree with the Cu–S_Z2_ distances in the present analysis (Fig. 3C), but are longer than typical water ligands to copper in the range of 1.9–2.1 Å. A structurally flexible or partly occupied S_Z2_ could have been interpreted as a bridging water at moderate resolution and without additional information from anomalous scattering.

### Further ion-binding sites in N_2_O reductase

**Fig. 4:**
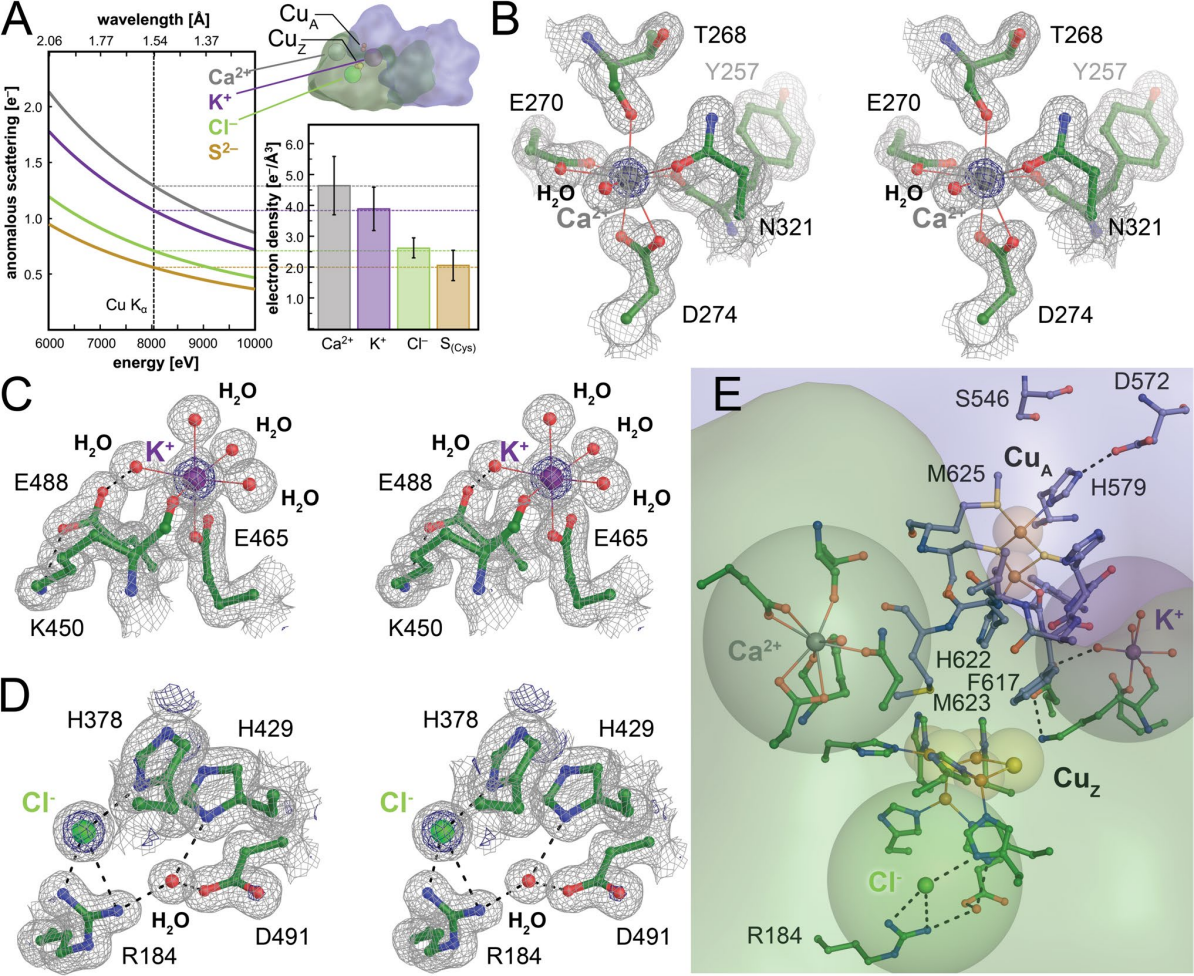
Additional ion-binding sites in *Mn*N_2_OR. **A** With the same number of electrons, Ca^2+^, K^+^, Cl^–^, and S^2–^ cannot be distinguished in 2*F*_o_–*F*_c_ difference electron density maps, but their anomalous scattering contribution at Cu K_∝_ energy differs slightly (left). The integrated anomalous difference density of the ion positions (right) scales very well with these expected differences. The depicted N_2_OR dimer highlights the positions of the ions and corresponds to panel (**E**). **B** Stereo representation of the Ca^2+^ site in the β-propeller domain, with the typical bidentate coordination by two carboxylate ligands. **C** Stereo rendering of the K^+^ site. The octahedral ligand field includes four waters, but only one carboxylate oxygen. **D** Stereo image of the Cl^−^-binding site located directly adjacent to Cu_Z_ [see (**E**)]. R184 as a positively charged ligands supports the assignment as an anion. All electron density maps are 2*F*_o_-*F*_c_ difference maps contoured at the 1σ (grey) and 5σ (blue) levels. **E** The relative positions of the ion sites with respect to the Cu_A_ and Cu_Z_ sites. Note that the K^+^ ion is placed in the dimer interface, while Ca^2+^ stabilizes a loop that clamps the β-propeller domain of protomer A to the cupredoxin domain of protomer B [56]

All known structures of N_2_O reductases contain additional ion-binding sites beside the copper centers Cu_A_ and Cu_Z_. Contrary to many other observations of surface-bound ions, these are specific sites, buried within the protein, and likely of functional importance. Although we only used diffraction data collected at a single energy, its high quality allowed us to evaluate the distinct anomalous scattering contribution of individual atoms as a means of identification. According to the Sasaki tables for anomalous scattering, the anomalous contribution of Ca^2+^ at the Cu K_α_ energy (8014 eV) is 1.29 e^−^, while that of K^+^ is 1.08 e^−^ [55], and there is a similar difference in the scattering contribution for Cl^–^ and S^2−^ (Fig. 4A). Note that all these ions are isoelectronic and therefore virtually indistinguishable in common difference electron density maps. The anomalous scattering signal is notoriously weak, but high data accuracy and a crystal form with four independent observations of each ion-binding site in the two dimers of the asymmetric unit allowed for a reliable evaluation according to the tabled values. For all sites, the refined B-factors agreed with their surroundings, indicating full occupancy (Fig. 4A).

The first site in the enzyme is a Ca^2+^ ion bound in the N-terminal β-propeller domain, connecting β-strands 2 and 3 of the fourth blade (Figs. 1D, 4B). With bound Ca^2+^, the loop contacts the C-terminal cupredoxin domain of the other monomer, stabilizing its interaction with the β-propeller, while in an apo-structure of *Shewanella denitrificans* N_2_OR, the loop was disordered in the absence of the cation [56]. Ca^2+^ binding raised the *T*_m_ of the protein, suggesting that this binding event occurs after the insertion of the copper sites to provide stability to the mature enzyme [56]. Interestingly, a recent structure of apo-N_2_OR bound to the copper site maturation complex NosDFY already had Ca^2+^ bound, although both copper centers were absent [57]. However, this structure was only obtained with an inactive E154Q variant of the ABC protein NosF that rendered the complex incompetent for copper uptake and transfer to N_2_OR. In the present analysis of *Mn*N_2_OR, the Ca^2+^ site was fully defined and showed a typical coordination environment that in essence was octahedral but included two bidentate ligands, E260 and D274. Further ligands to Ca^2+^ were the amide oxygen of N321, the backbone amide oxygens of residues Y257 and T268, and a water molecule (Fig. 4B).

In the initial structural models for N_2_OR from *P. denitrificans* and *M. nauticus*, a further ion-binding site was modeled as a second Ca^2+^ [14, 15, 17, 18]. This was contested in the analysis of *Ps*N_2_OR, where the conserved feature was interpreted as a coordinated K^+^ ion [25]. As calcium and potassium ions are not readily discriminated in an electron density map, the point of distinction made was the coordination environment that is the exact same in the present structure, showing a nearly undistorted octahedral geometry. Four of the six ligands to the ion were water molecules, and only one acidic residue, E465, acted as a monodentate ligand, compensating the charge of K^+^. The sixth ligand was the backbone carbonyl of the highly conserved K450, whose N_ζ_ amino group is close to Cu_Z_. It was suggested to play a functional role during catalysis, as a proton donor for the reaction [58–60] and in supporting conformational flexibility of the Cu_Z_ site [27] (Fig. 4C). The integrated anomalous scattering contribution for the Ca^2+^ sites exceeded that for K^+^ by exactly the expected amount (Fig. 4A). Together with the finding that upon Ca^2+^ reconstitution of the otherwise similar *S. denitrificans* N_2_OR, only one site is occupied by the divalent cation [56], we conclude that this site, as in *Ps*N_2_OR [25], binds K^+^ specifically. The K^+^ site is located on the surface of the Cu_Z_ domain, in the interface with the Cu_A_ domain of the other N_2_OR protomer (Fig. 4E). Although four of its ligands are water molecules, these are buried within the protein matrix of the dimer, stabilized by a tight hydrogen-bonding network and thus not readily exchangeable. The K^+^ site furthermore is in close (if indirect) contact with Cu_A_, via hydrogen bonds from water ligands, on one hand to the N_ε1_ atom of the side chain of W616 that coordinates Cu_A_ through its backbone carbonyl, and on the other hand to the backbone carbonyl of F617, a conserved residue that together with M623 controls access to the N_2_O binding site on Cu_Z_ [25]. A conserved and possibly functionally essential residue is E488 that forms a hydrogen bond to a water ligand of K^+^, but also to the backbone amide of F617. It also forms a salt bridge to the side chain of K450, orienting it near Cu_Z_ and providing a compensating charge (Fig. 4E). Taken together, F617, E488, and K450 form an efficient electron conduit between the Cu_A_ and the Cu_Z_ site. Alternatively, we have suggested that the exact same arrangement in *Ps*N_2_OR (residues F621, E492, and K454) acts as a proton conduit that runs along the Cu_A_ site and is gated by the conformational change of the distal histidine ligand to Cu_A_, H579 [52]. The present analysis of the *Mn*N_2_OR structure is fully in line with this. The second ion-binding site in N_2_OR thus holds a K^+^ ion that plays a very specific role in connecting the Cu_A_ and Cu_Z_ sites, integrating them into a single functional active site within N_2_OR. For N_2_OR assembly, this choice of ions is deliberate. Calcium binding locks the structure in a closed state that no longer allows for the insertion of copper during the maturation of the enzyme in the periplasm [56], while the position of K^+^ indicates that it already is inserted upon dimerization of apo-NosZ. This step occurs in the cytoplasm prior to the export of the enzyme as a folded dimer via the Tat system, and the K^+^ site is no longer accessible after the dimer has formed [56]. It is, therefore, important that the correct ion binds in the proper sequence in the right cellular compartment.

One additional ion-binding site was consistently observed in all structures of N_2_OR enzymes reported to date. Located close to the Cu_Z_ site, a significant electron density peak appeared that could not be modeled as water. Due to its position close to the positively charged R184 (*M. nauticus* numbering) and an electron density peak with approximately twice the magnitude of a water (after occupancy refinement), this feature was modeled as a chloride anion. Besides R184, the only other amino acid that directly ligated this ion was H378, a ligand to Cu_Z1_ (Fig. 4D). The role of this ion is unclear, although its charge may help in shaping the electrostatic environment of the Cu_Z_ site. Moreover, chloride is not easy to distinguish from the isoelectronic hydrosulfide anion, HS^−^, adding to the enigma of the possible flexibility of S_Z2_, as discussed above. The average anomalous signal peak intensity at this position is slightly higher than that for all cysteine sulfides in the structure, but as the varying *B*-factors of these amino acids in different parts of the structure play strongly into this measurement and the anomalous signal strength for chloride should only be 14% higher than for sulfide, the distinction is not a clear as for the case of K^+^ versus Ca^2+^. We therefore used only the most well-defined sulfur atoms in the structure, the cysteine S_γ_ atoms, but not the S_δ_ of methionines. Here, the sulfur anomalous signal perfectly coincided with the tabled values, while the four presumed Cl^−^ sites showed a stronger signal that corresponded to the Sasaki values for this element (Fig. 4A). Our analysis of the anomalous scattering properties of all ion sites supports an assignment as Cl^–^ rather than S^2–^, but its influence on the properties of Cu_Z_—if any—remains to be understood.

### Implications for the catalytic mechanism of N_2_OR

The electron-transferring Cu_A_ and the unique Cu_Z_ combine at a dimer interface to create an intricate active site for the reduction of inert N_2_O. The observation of two distinct conformations of Cu_A_ in the present analysis is in line with our earlier findings on *Ps*N_2_OR [25, 26], but it has not been reported for any of the multiple other descriptions of mixed-valent Cu_A_ sites. Most intriguingly, the precise definition of two conformations for Cu_A_ implies that the *C*_2_-symmetric N_2_OR dimer must pack in the crystal lattice in a non-random fashion, as otherwise the two conformations of the site should be randomly distributed, and the resulting electron density maps should represent an average of both conformations. We initially assumed that the IN- and OUT-conformations of Cu_A_ might lead to changes at the protein surface that could then affect crystal packing, but we could not identify any significant differences between the two protomers. Both the *P. stutzeri* and the present *M. nauticus* enzyme crystallized in the lowest-symmetry space group, triclinic *P*1, resulting in a rather simple crystal packing that may prevent that different conformations average out, so that it remains possible that this feature could not be discerned in other structures. Note also that the serine required for the OUT-conformation (S546 in *Mn*N_2_OR) is conserved in N_2_OR sequences, but not in the Cu_A_ domains of oxidases or the engineered sites in generic cupredoxins. Mechanistically, a gating mechanism for electron transfer to and through Cu_A_ is highly interesting. Cu_A_ is a single-electron transfer site, so that the 2-electron reduction of N_2_O to N_2_ should proceed in two consecutive reduction steps, possibly with an enzyme-bound intermediate, formally [N_2_O]^−^. In this case, the role of Cu_Z_ would be to bind and activate N_2_O as a prerequisite for electron transfer from Cu_A_. The finding that crystals of N_2_OR bind N_2_O between the Cu_Z_ and Cu_A_ sites upon pressurization with the gas is well in line with this model [25]. However, we so far failed to isolate and analyze a one-electron-reduced intermediate of the reaction, which should be formed if enzyme with reduced Cu_A_ was incubated with N_2_O in the absence of further reductant. In addition, N_2_OR exhibited higher catalytic activity when Cu_Z_ was reduced to an all-cuprous state with 4 Cu(I) [60]. Turnover from this highly reduced state led to a one-electron-oxidized form, designated Cu_Z_^0^. While at the same redox level as the Cu_Z_* state, only Cu_Z_^0^ could be reduced from Cu_A_ using a physiological reductant [60]. The mechanistic implication is that for the reduction of N_2_O, one electron is provided by Cu_Z_ and the other by Cu_A_. This results in a concerted 2-electron reduction, presumably without a long-lived intermediate. The observed transition from an OUT to an IN conformation upon N_2_O binding [25] may be key to this process, as the geometric changes reported here likely affect the midpoint potential for Cu_A_ and potentially serve as a trigger for electron transfer to the substrate.

The observed uniformity of the unique Cu_Z_ cluster indicates that the heterogeneity seen before at this site may indeed have been due to radiation damage or photoreduction processes during data collection. Available structures mostly differ in the ligand or ligands bridging Cu_Z1_ and Cu_Z4_, but not in the tetrahedral core formed by Cu_Z2-4_ and S_Z1_. Previously, this was interpreted as lability of S_Z2_ that was postulated to fully dissociate from the cluster to facilitate N_2_O binding as a bridging ligand to Cu_Z1_ and Cu_Z4_ [29, 61]. The H382A variant of *Ps*N_2_OR then revealed that the spectroscopic differences between form I and II of the enzyme, or the Cu_Z_ and Cu_Z_* form of the active site cluster, are also reproduced if S_Z2_ dissociates only from Cu_Z1_ to interact with the nearby lysine [27]. This flexibility can rationalize that S_Z2_ in many structures seems to have lower electron density (leading to an assignment as OH^−^) or even two distinct positions that were then interpreted as bound water. Further work will be required to confirm this hypothesis and deliberately populate the two conformational states for in-depth analysis. Also, in many cases, one or several of the histidine ligands to the copper ions are not ideally oriented toward the metal, indicating that the observed structures may be partially demetallated. This is another typical effect of radiation damage, and the structural uniformity of Cu_Z_ in the present analysis at low X-ray dose underscores the value of this approach for the analysis of sensitive metal sites in general and the intrinsically flexible Cu_Z_ in particular.

## Data Availability

The atomic coordinates and experimental structure factor amplitudes for the low-dose structure of *M. nauticus* N_2_OR have been deposited with the Protein Data Bank at http://www.pdb.org with the accession number 9F8X.

## Abbreviations

N_2_OR: Nitrous oxide reductase
EPR: Electron paramagnetic resonance
MR: Molecular replacement
